# Different Movement Strategies in the Countermovement Jump Amongst a Large Cohort of NBA Players

**DOI:** 10.3390/ijerph17176394

**Published:** 2020-09-02

**Authors:** Jacob Rauch, Eric Leidersdorf, Trent Reeves, Leah Borkan, Marcus Elliott, Carlos Ugrinowitsch

**Affiliations:** 1 Peak Performance Project, Santa Barbara, CA 93101, USA; eleidersdorf@p3.md (E.L.); treeves@p3.md (T.R.); lborkan@p3.md (L.B.); melliott@p3.md (M.E.); 2 Laboratory of Adaptations to Strength Training, Escola de Educação Física e Esporte, Universidade de Sao Paulo, São Paulo 05508-060, Brazil; ugrinowi@usp.br

**Keywords:** NBA, CMJ, biomechanics, 3-D motion capture, cluster analysis

## Abstract

Previous research has demonstrated large amounts of inter-subject variability in downward (unweighting & braking) phase strategies in the countermovement jump (CMJ). The purpose of this study was to characterize downward phase strategies and associated temporal, kinematic and kinetic CMJ variables. One hundred and seventy-eight NBA (National Basketball Association) players (23.6 ± 3.7 years, 200.3 ± 8.0 cm; 99.4 ± 11.7 kg; CMJ height 68.7 ± 7.4 cm) performed three maximal CMJs. Force plate and 3D motion capture data were integrated to obtain kinematic and kinetic outputs. Afterwards, athletes were split into clusters based on downward phase characteristics (*k*-means cluster analysis). Lower limb joint angular displacement (i.e., delta flexion) explained the highest portion of point variability (89.3%), and three clusters were recommended (Ball Hall Index). Delta flexion was significantly different between clusters and players were characterized as “stiff flexors”, “hyper flexors”, or “hip flexors”. There were no significant differences in jump height between clusters (*p* > 0.05). Multiple regression analyses indicated that most of the jumping height variance was explained by the same four variables, (i.e., sum concentric relative force, knee extension velocity, knee extension acceleration, and height) regardless of the cluster (*p* < 0.05). However, each cluster had its own unique set of secondary predictor variables.

## 1. Introduction

The countermovement jump (CMJ) is an essential motor skill for a variety of sports including volleyball, soccer and basketball. For basketball in particular, CMJ performance may provide a technical/tactical edge in both offensive and defensive actions (i.e., improving the probability of effective shots, rebounds and blocks) [1]. Though the demands of the game will likely impose temporal restrictions to the athlete’s jump, for standardization purposes a CMJ with no time constraints is included as an assessment at the National Basketball Association (NBA) combine, which is regarded as the top level of basketball competition in the world [2,3]. Furthermore, due to its importance for a variety of sports, the CMJ is arguably the most common assessment implemented to measure lower body-ballistic performance [4]. Thus, determining the variability in CMJ movement strategy may be more informative to coaches and athletes than jumping height per se, as the former may be associated with the ability of the players to perform specific technical and tactical skills. 

A defining feature of the CMJ is the downward phase performed prior to push off. According to McMahon et al., (2018) [5], the downward phase is composed of two components, unweighting and braking. Jumps with a downward phase typically result in greater jump heights (2–4 cm) compared to jumps with no downward phase (i.e., squat jumps) [6]. Interestingly, researchers have identified differences in movement strategies between individuals during the downward phase [7,8,9,10]. Different downward phase movement strategies are relevant, as the velocity and range of motion of the lower limb joints during the downward phase can affect the efficiency of the stretch shortening cycle (SSC) and concentric force potentiation, and thus CMJ performance [11,12,13,14].

It has been assumed that elite athletes are able to self-select their movement strategies in order to maximize jump height [15,16,17]. In regard to elite basketball players, research has shown different, but consistent, downward phase strategies between and within players, respectively [13]. These findings suggest that each athlete has a unique and consistent jumping style when there is no time constraint in which to perform the jump. However, the few available studies examining downward phase variables within elite basketball players had small sample sizes (i.e., *n* = 11) [13,17], hampering the ability to determine recurrent jumping patterns. In addition, a large sample size of top-level athletes (i.e., NBA players) offers the ability to cluster jumping strategies, which may improve the precision of determining the effects that different movement strategies have on temporal, kinematic and kinetic CMJ variables. Some of the most common temporal, kinematic and kinetic variables related to CMJ performance include multi-segment coordination, vertical ground reaction force, joint flexion velocities and joint extension velocities [18,19].

Therefore, the purpose of this investigation was two-fold: (1) to determine possible differences in downward phase kinematics of the CMJ in 178 NBA players through a cluster analysis; and (2) to determine the temporal, kinetic and kinematic variables that are associated with different downward phase movement strategies.

## 2. Materials and Methods

### 2.1. Experimental Design

The present study used a cross-sectional design to examine the downward phase movement pattern of NBA players in the CMJ. Every athlete agreed to release their data and was on an active roster in the NBA at the time of data collection. Standard biomechanical procedures for analyzing the CMJ were implemented. Afterwards, athletes were clustered based on similarities in movement strategy during the downward phase of the CMJ. Downward phase movement strategies were evaluated through the following parameters: delta flexion (i.e., lower limb joint angular displacement), maximum flexion, average flexion velocity and maximum flexion velocity at the ankle, knee and hip joints, respectively. These variables were selected as they account for the rate and range traveled at each joint, which impact the efficiency of the SSC [11,12,20,21]. The downward phase variable which best explained the point variability was used to form the final clusters. Lastly, stepwise regressions were run on the entire data set and individual clusters to examine if the clusters had different predictor variables for CMJ performance.

### 2.2. Subjects

One hundred and seventy-eight professional NBA players (mean ± standard deviation (SD): age 23.6 ± 3.7 years, height 200.3 ± 8.0 cm; mass 99.4 ± 11.7 kg; CMJ height 68.7 ± 7.4 cm) volunteered to participate in the study. To be eligible for inclusion, all subjects had to be on an active NBA roster and free from any musculoskeletal injuries at the time of data collection. All subjects signed an informed consent form going over the details of the procedures and inherent risks.

### 2.3. Procedures

Before testing, athletes underwent a standardized warm-up which consisted of a series of 16 dynamic movements performed on a twenty-yard indoor track. Movements included lunge and hinge variations and progressed to low intensity plyometric exercises. After the warmup, subject’s weight, (Bertec Corporation, Columbus, OH, USA) overhead reach, (Vertec, Sports Imports, Hilliard, OH, USA) standing height, and playing position were recorded by the principle investigator. Subjects were given practice trials followed by three maximal attempts, interspersed by ninety seconds. Subjects were instructed to jump as high as possible and knock away as many of the half inch veins as they could on the Vertec. Countermovement velocity and depth were self-selected. The jump with the greatest vertical displacement was used for further analysis.

A nine-camera Simi (Simi Reality Motions Systems GmbH, Unterschleissheim, Germany) Motion analysis system in conjunction with dual Bertec (Model No. 6090, Bertec Corporation, Columbus, OH, USA) force platforms were integrated to collect jump data. A twenty lower extremity reflective marker set was used. Markers were placed on the first metatarsal joint, medial and lateral malleolus, heel, shank, medial and lateral condyles of the femur, anterosuperior iliac spines, greater trochanters, fourth lumbar vertebrae, and the sternum. The motion capture system synchronized motion (120 Hz) and force (1000 Hz) data. Identified marker mergences were split manually and gaps were reconstructed with a cubic spline interpolation for a maximum gap size of 20 frames [22]. Raw data were then filtered using a second order low pass digital filter (Frequency: 10 Hz) and standard inverse kinematics were followed [23]. Final data points were analyzed and exported with MATLAB (MATLAB, The MathWorks, Inc., Natick, MA, USA).

#### Data Analysis

The following variables were used for further analysis: body weight, jump height, net relative impulse, relative un-weighting force, sum (left and right) braking force, relative sum (left and right) concentric force, total movement time, maximum joint flexion average, delta joint flexion, joint total range of motion, maximal joint flexion velocity, joint flexion acceleration, joint extension, joint extension velocity, joint extension acceleration, and time to maximum joint flexions and extensions. All kinetic variables were obtained from force plate vertical axis data and followed the methodological recommendations described by McMahon et al. (2018) [5]. In brief, bodyweight was calculated as the 5 s average of the vertical force while the athlete was standing still on a single force plate. Body mass was obtained by dividing the bodyweight by acceleration due to gravity. Jump height was calculated by subtracting the maximum height reached on the Vertec (in) from the standing reach (in) and converting it to cm. Net relative impulse (N·s/kg^−1^) was defined as the integral of the force time curve divided by the subject’s body mass. Total movement time was calculated by subtracting the takeoff instant (vertical force of ≤10 N) from the initiation of the countermovement, which was determined as a drop in vertical force of 2 SD below average body weight. Peak relative unweighting force refers to the first local minimum (Fz) following the initiation of the countermovement [5] divided by body mass (kg). Sum braking and concentric relative forces were calculated by taking the sum of the peak vertical force (Fz) values obtained from the left and right force plates during the braking and concentric phases respectively and dividing those values by body mass (kg). The downward phase began with the initiation of the countermovement and ended once vertical velocity equaled zero. The concentric phase was determined by the initiation of a positive center of mass velocity until the instant of take off as evidenced by vertical force values of ≤10 N. Inverse kinematics were used to export maximum joint flexions, delta flexion, joint total range of motion, maximum joint flexion velocity, joint flexion acceleration, joint extension velocity, joint extension acceleration, and time to maximal joint flexions and extensions [24]. Maximum joint flexions were determined as the maximum flexion angle achieved at each joint. Time to maximal joint flexions was determined by subtracting the time of peak joint flexion from the initiation of the countermovement. Time to joint extensions was determined by subtracting the time of which peak joint extensions occurred from the initiation of the countermovement. Delta joint flexion was defined as the total range traveled from the movement initiation until peak flexion. Joint total range of motion was determined by taking the degrees of motion traveled from peak flexion to peak extension [24]. Peak joint flexion velocities and accelerations were determined by taking the derivative of the position-time and velocity-time curves for each joint respectively during the downward phase. Peak joint extension velocities and accelerations were determined by taking the derivative of the position-time and velocity-time curves for each joint respectively during the concentric phase. The MATLAB code which exported each of these variables was developed in partnership with the motion analysis company (Simi Reality Motions Systems GmbH, Unterschleissheim, Germany). Prior to data collection an inhouse rep to rep reliability study was performed on 12 subjects with similar jumping abilities as the subjects included in the investigation.

### 2.4. Statistical Analysis

All temporal, kinetic and kinematic data were normalized (centered and scaled) prior to analysis, a common practice in clustering and regression modeling [25]. A *k*-means cluster analysis was run with four sets of downward phase kinematic variables: delta flexion, maximum flexion angles, average flexion velocity and maximum flexion velocity at the ankle, knee and hip respectively. It should be noted that a considerable challenge facing cluster analysis is choosing the optimal variables, and number of variables, to create the clusters. We attempted to account for this by analyzing the aforementioned sets of downward phase kinematic variables independently and selecting the set of variables which created the most stable clusters. While downward phase movement strategies can be altered by both velocity and range, we decided to only account for one set of variables at a time as adding in additional variables may reduce the stability of the cluster. The ball hall index was used to determine the optimal number of clusters. Afterwards, a one-way analysis of variance (ANOVA) was run to test for between-cluster differences for each dependent variable. A one-way ANOVA was also run on descriptive characteristics (CMJ height, net relative impulse, body weight, height, relative concentric force, relative braking force, joint extension velocities and total movement time (TMT)) to examine if there were any between-cluster differences in common CMJ variables. Additionally, a one-way ANOVA was run comparing the average position between clusters (categorically coding guards = 1, forwards = 2, and centers = 3). Whenever a significant F-value was obtained, a post hoc test with a Tukey adjustment was performed for multiple comparison purposes. Finally, a stepwise regression was performed on the entire data set and for each individual subgroup, having jumping height as the dependent variable. The Bayesian Information Criterion (BIC) index was used to determine which variables were included in the final model. For each regression model, variables are listed with their partial and total adjusted *R*^2^ values. Inverse relationships are depicted by an *. To account for co-linearity between variables, a variance inflation factor (VIF) was run. For a variable to be included in the final output the VIF had to be below 10.0. Reliability was assessed using intraclass correlation coefficients (ICCs). Based on the recommendations of previous literature, obtaining an ICC of >0.70 was set as a minimum acceptable reliability [26]. Data were analyzed using R-studio (RStudio Team (2015). RStudio: Integrated Development for R. RStudio, Inc., Boston, MA, USA) version 1.1.49. Significance was set as *p* < 0.05, and data were presented as mean and standard deviation, delta difference, percent difference, and Cohens d effect size utilizing the pooled SD.

## 3. Results

### 3.1. Reliability

The ICCs for each kinematic and kinetic variable included in the regression models can be found below. Kinematic ICC values for each variable are listed from the hip, knee and ankle respectively: maximum joint flexion average (0.76, 0.90, 0.92), delta joint flexion (0.89, 0.80, 0.73), joint total range of motion (0.70, 0.88, 0.93), maximum joint flexion velocity (0.90, 0.92, 0.85), maximum joint flexion acceleration (0.86, 0.86, 0.76), maximum joint extension (0.92, 0.97, 0.98), maximum joint extension velocity (0.71, 0.91, 0.92), maximum joint extension acceleration (0.77, 0.79, 0.93). The ICCs for the kinetic variables are as follows; sum braking force (0.91), relative unweighting force (0.97), sum concentric relative force (0.91), total movement time (0.97).

### 3.2. k-Means Cluster Analysis

**Figure 1:**
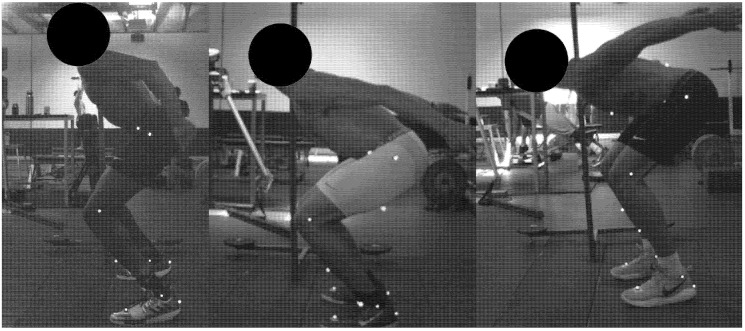
Example of athlete from cluster 1,2 and 3 respectively. Example was taken from peak braking force during the countermovement jump.

Delta flexion explained the highest portion of the point variability (89.43%) compared to the other three downward phase variables. Three subgroups explained most of the variance in the data according to the Ball Hall Index. The degree of delta flexion at the ankle, knee and hip joints within each cluster can be found in Table 1 and an example athlete from each cluster can be found in Figure 1.

### 3.3. One Way Analysis of Variance (ANOVA) Delta Flexion at Each Joint between Subgroups

Cluster 1 demonstrated significantly less delta ankle dorsi flexion compared to Cluster 3 (∆−1.4°, % Difference (Diff) −10.1, Effect Size (ES) 0.43, *p* = 0.041) and Cluster 2 (∆−8.3°, % Diff −48.1, ES 2.52, *p* = 0.000). Furthermore, Cluster 2 demonstrated significantly greater delta ankle dorsi flexion compared to Cluster 3 (∆6.9°, % Diff 38.4, ES 2.08, *p* = 0.000). For delta knee flexion, Cluster 1 had significantly lower range of motion compared to Cluster 2 (∆−18.8°, % Diff −37.4, ES 2.55, *p* = 0.000) and Cluster 3 (∆−9.8°, % Diff −21.4, ES 1.33, *p* = 0.000). Additionally, Cluster 2 traveled through more range compared to Cluster 3 (∆9°, % Diff 16.3, ES 1.23, *p* = 0.000). For delta hip flexion, Cluster 1 traveled through the least amount of range compared to Cluster 3 (∆−28.8°, % Diff −71.5, ES 2.42, *p* = 0.000) and Cluster 2 (∆−24.1, % Diff −63.5, ES 2.03, *p* = 0.000). However, Cluster 3 and Cluster 2 were not significantly different from each other, (∆4.7°, % Diff 9.0, ES 0.39, *p* = 0.12).

### 3.4. One Way Analysis of Variance (ANOVA) Descriptive Characteristics between Clusters

The complete results for the one-way ANOVA on descriptive characteristics between clusters can be found in Table 2. In brief, there were no statistical differences between clusters for CMJ height, net relative impulse, body mass, height and maximum plantar flexion velocity (*p* > 0.5). For maximum knee extension velocity, Cluster 2 demonstrated significantly greater outputs compared to Cluster 1 (∆40°·s^−1^, % Diff 4.8, ES 0.64, *p* = 0.001). Furthermore, there were no statistical differences between Cluster 3 and Cluster 1 (∆12.7°·s^−1^, % Diff 1.5, ES 0.20, *p* = 0.491), or Cluster 3 and Cluster 2 (∆−27.3°·s^−1^, % Diff −3.2, ES 0.44, *p* = 0.072). For concentric relative force Cluster 1 demonstrated significantly greater values compared to Cluster 2 (∆3.1 Fz·kg^−1^, % Diff 10.5, ES 1.06, *p* = 0.000) and Cluster 3 (∆2.4 Fz·kg^−1^, % Diff 8.0, ES 0.82, *p* = 0.000). However, there were no statistical differences between Cluster 3 and Cluster 2 (∆0.71 Fz·kg^−1^, % Diff 2.5, ES 0.24, *p* = 0.44). For total movement time Cluster 1 had the shortest time compared to Cluster 2 (∆−0.21 s, % Diff −25.4, ES 0.97, *p* = 0.00) and Cluster 3 (∆−0.26 s, % Diff −30.6, ES 1.23, *p* = 0.000). However, there were no statistical differences between Cluster 3 and Cluster 2 (∆0.05 s, % Diff 5.2, ES 0.26, *p* = 0.08).

### 3.5. One Way Analysis of Variance (ANOVA) Positional Distribution between Clusters

The one-way ANOVA results demonstrated that there were no statistical differences in the average position number between Cluster 1 and Cluster 2 (∆0.09, % Diff 5.8, ES 0.10, *p* = 0.76) as well as Cluster 1 and Cluster 3 (∆−0.27, Diff −15.6, ES 0.43, *p* = 0.079). However, the average position number in Cluster 3: (1.87 ± 0.71) was significantly greater compared to Cluster2: (1.51 ± 0.65) (∆0.36, % Diff 21.3, ES 0.54, *p* = 0.028). Further, we can infer that cluster three was composed of more forwards and centers compared to cluster two (Table 3).

### 3.6. Stepwise Regression

The stepwise regression results are presented in Table 4. Inverse relationships are depicted by an *. In brief, most of the predictor variables were similar between clusters sharing sum concentric relative force, knee extension velocity, knee extension acceleration, and height. Furthermore, Cluster 1 demonstrated negative relationships with max knee flexion and max plantar flexion. Cluster 2 demonstrated positive relationships with the time between the percentage of movement in which peak hip flexion and knee flexion occurred and delta knee flexion. Lastly, Cluster 3 demonstrated a positive relationship with hip total range of motion.

## 4. Discussion

The main purpose of this investigation was to examine the downward phase kinematics of a large subset of NBA players. Our main findings demonstrate that the angular displacement at each joint (i.e., delta flexion) during the downward phase can create three stable clusters. Cluster 1 (*n* = 77) “stiff flexors” travel through the least angular displacement at each joint. Cluster 2 (*n* = 49) “hyper flexors” travel through an above average angular displacement at each joint. Cluster 3 (*n* = 52) “hip flexors” travel through an average angular displacement at the ankle and knee joints but above average at the hip joint (Table 1). Although the total range traveled by stiff flexors (79.87 ± 14.52°) is significantly less compared to hyper flexors (131.2 ± 19.06°) the relative contribution of each joint is fairly similar between the two clusters (Stiff Flexors—Hip 32.45%, Knee 51.16%, Ankle 16.39%: Hyper Flexors—Hip 38.14%, Knee 45.54%, Ankle 16.32%). This is not the case for the hip flexors, as these athletes demonstrated a greater contribution of the hip joint compared to the other two clusters (Hip Flexors—Hip 45.61%, Knee 42.26%, Ankle 12.11%). We also determined the mechanical variables associated with different downward phase strategies. Our findings demonstrate that the same four variables (sum concentric relative force, knee extension velocity, knee extension acceleration, and height) explain 85.71%, 77.27% and 69.23% of the *R^2^* value between stiff flexors, hyper flexors and hip flexors respectively. Yet we found that secondary, “cluster specific”, variables associated with jumping height were mechanically related to the downward phase pattern.

Our findings are in agreement with Kipp et al., (2020) [17] who examined the kinetic and kinematic differences between 11 National Collegiate Athletic Association (NCAA) Division 1 male basketball players as they scaled from sub maximal to maximal CMJs. Through a single-subject analysis these researchers demonstrated a large between athlete variability in joint specific movement strategies implemented to achieve maximal CMJ height. Interestingly, these researchers also identified a subset of jumpers they referred to as hip dominant jumpers, primarily composed of forwards and centers who scale the work done at the hip joint as they move towards maximal CMJ performance. These findings partially corroborate with our hip flexor cluster as this cluster was composed of a greater percentage of forwards and centers (69.23%) compared to hyper flexors (42.85%) and stiff flexors (53.35%) (Table 3). Notably, our findings also demonstrate that when allowed to self-select the movement strategy to jump as high as possible elite basketball players can achieve similar jump heights irrespective of their downward phase strategy (Cluster1: 68.60 ± 6.55, Cluster2: 70.47 ± 9.17, Cluster3: 67.31± 6.35cm). However, our data does not provide any insight on how athletes from each cluster would change their downward phase patterns when facing temporal and spatial constraints imposed by game like scenarios. Thus, it would be pertinent for future investigations to determine how game like constraints impact downward phase patterns and CMJ performance amongst these clusters. Furthermore, the jump heights reported in our investigation are greater than the average jump heights reported by Kipp et al., (2020) [17] (CMJ Height: 62.4 ± 5.4 cm). There are two possible explanations for the aforementioned differences in jump height. (1) Kipp et al., (2020) [17] did not have an overhead goal while, in our sample, jump height was calculated as the reaching height difference between standing and jumping. (2) We tested top level professional athletes and this discrepancy between collegiate and professional athletes is expected.

From a physics standpoint, CMJ height is determined by the net vertical impulse (force x time integral) produced during the upward concentric phase [16,27]. As jump height is similar between clusters, net impulse between strategies is also comparable (Table 2). However, the means by which these impulses are generated is likely to differ between clusters. For example, stiff flexors may achieve high impulses through producing large forces over a short period of force application; whereas hyper flexors and hip flexors take more time to complete a jump, relying on an increased duration of force application. Therefore, each movement strategy appears to have its own method for developing large impulses.

Regarding the mechanical variables associated with different downward phase strategies, our findings demonstrate that the same four variables (sum concentric relative force, knee extension velocity, knee extension acceleration, and height) account for 85.71%, 77.27% and 69.23% of the adjusted *R^2^* value between stiff flexors, hyper flexors and hip flexors, respectively. Nevertheless, it is interesting to point out that each cluster has its own set of secondary, “cluster specific”, variables which also contribute to the adjusted *R^2^* values. For example, stiff flexors have a negative relationship with maximal plantar flexion, maximal knee extension, and amount of time between peak ankle and knee flexion. Collectively, these additional variables account for 14.29% of the adjusted *R^2^* value within stiff flexors. This suggests that stiff flexors cannot optimize jumping height when they increase the angular displacement at the knee and ankle joints. On the contrary, hyper flexors have a positive relationship with delta knee flexion, and amount of time between peak hip and knee flexion. These additional variables account for 22.73% of the adjusted *R^2^* value within hyper flexors and suggest that, to optimize jumping height in this cluster, players demonstrate high angular displacement at the knee and take more time between peak hip and knee flexion. Lastly, hip flexors demonstrate a positive relationship with hip total angular displacement, and a negative relationship with maximum knee extension, ankle angular displacement and time of movement in which peak dorsi flexion occurs. These secondary variables account for 30.77% of the *R^2^* value within hip flexors, suggesting that hip flexors go through more range at the hip, less extension at the knee and reach peak dorsi flexion earlier on in the movement while limiting total range at the ankle. Though these additional variables do not carry as much impact as the four variables which are consistent between clusters, they provide further support for clustering athletes based on similarities in movement strategies, as this type of analysis provides additional insights into cluster specific predictor variables that would have otherwise been washed out in a single group analysis.

Furthermore, as the net vertical impulse is what ultimately determines jump height, researchers have spent decades investigating the bodily strategies, temporal, kinetic and kinematic variables that are associated with high impulses [18,19,28]. It is worth noting however, that in addition to large amounts of inter-subject variability in downward phase strategies, researchers have also demonstrated large amounts of inter-subject variability in patterns of force application [29,30,31], thus, making it difficult to determine the precise impact that each of these variables may have on CMJ performance from traditional single group analysis, or small samples, which most of the available literature is comprised of. Nevertheless, for the entire data set, our regression results are in agreement with previous researchers who identified sum concentric relative force (i.e., VGRF), knee extension velocity, knee extension acceleration and height as significant predictors for CMJ performance [17,18,19,24,28].

Our study has several inherent limitations. Firstly, jump height was determined based on the vertex reaching height rather than based on vertical ground reaction force data. The readership should be aware that reaching to a vertex may introduce some bias into the measurement (e.g., shoulder elevation); however, contrary to untrained cohorts, our sample was composed of high level athletes that perform jump reaching tests routinely (i.e., high reliability) [3]. Furthermore, athletes were not given any time constraints when performing the CMJ, which limits our ability to generalize these findings into game like scenarios where temporal and spatial restrictions are imposed. Lastly, athletes came into the facility at various time points in their off-season and as a result we were not able to control for their physical state or previous jump training at the time of their assessment.

## 5. Conclusions

Our main findings demonstrate that elite basketball players tend to fall into one of three downward phase movement strategies during the CMJ: stiff flexors, hyper flexors, and hip flexors. Despite having significantly different downward phase strategies, athletes from each cluster are capable of producing similar jump heights. That said, the downward phase strategy does significantly impact how these jump heights are achieved. While the main jump height predictor variables for each cluster are similar, subtle differences between clusters do exist. Practitioners can utilize the specific information presented in this manuscript to develop a better understanding of how their athlete’s downward phase strategy affects common CMJ variables. Furthermore, the data presented can serve as a reference point for CMJ outputs at the highest level of basketball competition in the world. The descriptive nature of this study makes precise training recommendation difficult; however, practitioners may consider adding auxiliary exercises targeting some of the cluster specific predictor variables. One example may be adding half squats to stiff flexors training programs as increasing ability to produce force over minimal amounts of knee flexion may increase CMJ performance for this cluster. Another example may be adding additional posterior chain work for hip flexors as increasing the ability to produce force while traveling through a greater range of motion at the hip joint may increase CMJ performance for this cluster. In certain settings it may also be pertinent for practitioners to target kinetic/kinematic qualities that athletes in a given cluster may lack relative to other clusters. For example, as hyper flexors generally possess lower relative concentric force outputs, practitioners may incorporate general strength exercises into their programs targeting force development. Lastly, it is also important to address the demands of the game and the fact that if two athletes are competing for the same ball athletes who can A) better position themselves prior to performing the jump and B) perform the jump the fastest are likely to achieve a greater percentage of successful outcomes. Future research should look into whether these clusters have any correlation with game derived statistics, and may also attempt to determine if the differences in downward phase strategies reported herein are related to limited active range of motion in a specific joint, weakness of a given muscle group producing torque around a lower limb joint or demonstrate any relationship with prospective/ retrospective injuries.

## Figures and Tables

**Table 1:** Angular displacement at the ankle, knee and hip joints in clusters 1, 2 and 3 (mean ± standard deviation).

Cluster	*n*	Delta Ankle Dorsi Flexion (°)	Delta Knee Flexion (°)	Delta Hip Flexion (°)
1	77	13.1 *^,+^ ± 3.7	40.9 *^,+^ ± 7.8	25.9 *^,+^ ± 9.71
2	49	21.4 ^#,+^ ± 3.2	59.7 ^#,+^ ± 7.2	50.0 ^#^ ± 13.7
3	52	14.5 ^#,^* ± 2.6	50.7 ^#,^* ± 6.6	54.7 ^#^ ± 12.4
Total	178	15.8 ± 4.8	48.9 ± 10.7	41.0 ± 17.7

* Significantly different from group 2; ^+^ Significantly different from group 3; ^#^ Significantly different from group 1;

**Table 2:** Cluster Descriptive Characteristics.

Cluster	Plantar Flex Velo (m·s^−1^)	Knee Ext Velo (m·s^−1^)	Hip Ext Velo (m·s^−1^)	Rel Con Force (Fz·kg^−1^)	Rel Brk Force (Fz·kg^−1^)
1	737.68 ± 93.65	813.98 * ± 56.78	499.24 * ± 83.20	30.79 *^,+^ ± 3.05	22.69 ^+^ ± 4.34
2	760.87 ± 87.06	853.97 ^#^ ± 67.55	548.08 ^#^ ± 90.56	27.71 ^#^ ± 2.93	21.75 ^+^ ± 3.12
3	730.09 ± 93.21	826.67 ± 62.31	515.07 ± 83.91	28.42 ^#^ ± 2.49	19.92 ^#,^* ± 3.01
Total	741.85 ± 92.55	828.70 ± 63.69	517.31 ± 87.82	29.25 ± 3.17	21.62 ± 3.85
**Cluster**	**Net Rel Impulse (N·s·kg^−1^)**	**TMT (s)**	**Height (cm)**	**Weight (kg)**	**CMJ Height (cm)**
1	3.27 ± 0.33	0.72 * ± 0.18	199.97 ± 8.08	99.45 ± 11.49	68.60 ± 6.55
2	3.26 ± 0.38	0.93 ^#^ ± 0.22	198.63 ± 7.46	97.11 ± 11.98	70.47 ± 9.17
3	3.19 ± 0.29	0.98 ± 0.22	202.18 ± 7.86	101.52 ± 11.50	67.31 ± 6.35
Total	3.24 ± 0.33	0.85 ± 0.24	200.25 ± 7.96	99.41 ± 11.75	68.74 ± 7.41

^#^ Significantly different from group 1; * Significantly different from group 2; ^+^ Significantly different from group 3. Plantar Flexion Velocity (Plantar Flex Velo); Knee Extension Velocity (Knee Ext Velo); Hip Extension Velocity (Hip Ext Velo); Relative Concentric Force (Rel Con Force); Relative Braking Force (Rel Brk Force); Net Relative Impulse (Net Rel Impulse); Total Movement Time (TMT); Countermovement Jump Height (CMJ Height).

**Table 3:** Absolute and Relative Positional Distribution of the players within clusters and total.

Cluster	*n*	Guards Total	Guards %	Forwards Total	Forwards %	Centers Total	Center %
1	77	39	50.65	26	33.77	12	15.58
2	49	28	57.14	16	32.65	5	10.20
3	52	16	30.77	24	46.15	12	23.08
Total	178	82	46.07	67	37.64	29	16.29

**Table 4:** Variables included in the multiple regression equation and individual r values within each cluster predicting the jumping height.

Cluster	Con Rel Force	Knee Ext Velo	Max Knee Flex *	Knee Ext Accel	Height *	Max Plantar Flex *	PAKT		*R* ^2^
1	0.3	0.46	0.49	0.54	0.57	0.61	0.63		0.63
**Cluster**	**Knee Ext Velo**	**Con Rel Force**	**Max Plantar Flex**	**Knee Ext Accel**	Height *	**PHKT**	PTT Dorsi *	**Delta Knee Flex**	***R*** ^**2**^
2	0.39	0.56	0.67	0.71	0.79	0.81	0.83	0.88	0.88
**Cluster**	**Knee Ext Velo**	Max Knee Ext *	**Con Rel Force**	Height *	Ankle ROM *	**Hip ROM**	**Knee Ext Accel**	PTT Dorsi *	***R*** ^**2**^
3	0.36	0.5	0.6	0.64	0.69	0.72	0.76	0.78	0.78
**Total**	**Knee Ext Velo**	**Con Rel Force**	Max Knee Flex *	Max Plantar Flex *	Height *	**Max Hip Flexion**			***R*** ^**2**^
	0.34	0.49	0.57	0.61	0.66	0.69			0.69

Row 1—Concentric relative force, knee extension velocity, maximum knee flexion, knee extension acceleration, height, maximum plantar flexion, percentage difference of the time through the movement between peak ankle dorsi-flexion and peak knee flexion (PAKT). Row 2—Knee Extension velocity, concentric relative force, maximum plantar flexion, knee extension acceleration, height, percentage difference of the time through the movement between peak hip and knee flexion (PHKT), percentage of the movement in which peak dorsi flexion occurs (PTT). Row 3—Knee Extension velocity, maximum knee extension, concentric relative force, height, hip total range of motion (ROM), knee extension acceleration, percentage of the movement in which peak dorsi flexion occurs. Row 4—Knee Extension velocity, concentric relative force, maximum knee flexion, maximum plantar flexion, height, maximum hip flexion. * Indicates negative relationship with variable.
